# Transcriptome analysis combined with Mendelian randomization screening for biomarkers causally associated with diabetic retinopathy

**DOI:** 10.3389/fendo.2024.1410066

**Published:** 2024-07-03

**Authors:** Junyi Liu, Jinghua Li, Yongying Tang, Kunyi Zhou, Xueying Zhao, Jie Zhang, Hong Zhang

**Affiliations:** ^1^ Department of Ophthalmology, The Second Affiliated Hospital of Kunming Medical University, Kunming, China; ^2^ Department of Ophthalmology, Dali Bai Autonomous Prefecture People’s Hospital, Dali, China

**Keywords:** diabetic retinopathy, enrichment analysis, immune infiltration, Mendelian randomization, regulatory network

## Abstract

**Background:**

Diabetic retinopathy (DR) is considered one of the most severe complications of diabetes mellitus, but its pathogenesis is still unclear. We hypothesize that certain genes exert a pivotal influence on the progression of DR. This study explored biomarkers for the diagnosis and treatment of DR through bioinformatics analysis.

**Methods:**

Within the GSE221521 and GSE189005 datasets, candidate genes were acquired from intersections of genes obtained using WGCNA and DESeq2 packages. Mendelian randomization (MR) analysis selected candidate biomarkers exhibiting causal relationships with DR. Receiver Operating Characteristic (ROC) analysis determined the diagnostic efficacy of biomarkers, the expression levels of biomarkers were verified in the GSE221521 and GSE189005 datasets, and a nomogram for diagnosing DR was constructed. Enrichment analysis delineated the roles and pathways associated with the biomarkers. Immune infiltration analysis analyzed the differences in immune cells between DR and control groups. The miRNet and networkanalyst databases were then used to predict the transcription factors (TFs) and miRNAs, respectively, of biomarkers. Finally, RT-qPCR was used to verify the expression of the biomarkers *in vitro*.

**Results:**

MR analysis identified 13 candidate biomarkers that had causal relationships with DR. The ROC curve demonstrated favorable diagnostic performance of three biomarkers (*OSER1*, *HIPK2*, and *DDRGK1*) for DR, and their expression trends were consistent across GSE221521 and GSE189005 datasets. The calibration curves and ROC curves indicated good predictive performance of the nomogram. The biomarkers were enriched in pathways of immune, cancer, amino acid metabolism, and oxidative phosphorylation. Ten immune cell lines showed notable disparities between the DR and control groups. Among them, effector memory CD8+ T cells, plasmacytoid dendritic cells, and activated CD4+ T cells exhibited good correlation with biomarker expression. The TF-mRNA-miRNA network suggested that hsa-mir-92a-3p, *GATA2*, and *RELA* play important roles in biomarker targeting for DR. RT-qPCR results also demonstrated a notably high expression of *HIPK2* in patients with DR, whereas notably low expression of *OSER1*.

**Conclusion:**

*OSER1*, *HIPK2*, and *DDRGK1* were identified as biomarkers for DR. The study findings provide novel insights into the pathogenesis of DR.

## 1 Introduction

Diabetic retinopathy (DR) is a disease that causes vision loss in adults aged 20 to 74 years (1). The incidence of visual impairment and blindness caused by DR in low- and middle-income countries has increased significantly due to the increasing incidence of type 2 diabetes (2). About one-third of the 260 million people with diabetes have DR, and one-third of these patients are diagnosed with advanced DR or diabetic macular edema, and most of these patients have severe vision loss and a serious impact on their quality of life (3). In China, the incidence of diabetes is steadily increasing, with projections indicating that by 2045, approximately 174 million individuals will be diagnosed with diabetes (4). The diagnosis of DR mainly depends on history of diabetes and changes in the fundus of the eye as assessed by fundus photography, optical coherence tomography, and fundus fluorescence angiography (5). At present, DR is diagnosed solely based on the clinical manifestations, and the corresponding symptomatic treatment is based on the findings of fundus evaluation, but there is a lack of predictive and effective evaluation methods for DR. Among the many risk factors for DR, the most relevant factors are diabetes progression and poor glycemic control (6). The pathogenesis of DR is complicated, involving multiple molecular and biochemical mechanisms related to the homeostasis of retinal blood vessels and cells. The treatment for DR mainly includes intravitreous drug injection and retinal laser photocoagulation (7)Due to our lack of understanding of DR pathogenesis, there is also a lack of effective clinical treatment options. Therefore, it becomes vital to identify robust biomarkers for DR and further investigate the mechanisms underlying DR pathogenesis. The ideal therapeutic strategy for the clinical identification and management of DR should aim to enhance the patients’ quality of life to the fullest.

Mendelian randomization (MR) is a novel epidemiological design tool that follows the genetic law of random distribution of alleles from parents to offspring (8). This tool has been increasingly used for establishing causal relationships between exposure factors and disease risks, made possible through advancements in statistical techniques, availability of extensive datasets, progress in epigenetics research, and the emergence of various ‘omics’ technologies (9). Therefore, in this study, we used MR to screen for biomarkers that exhibit a causal relationship with DR.

**Figure 1:**
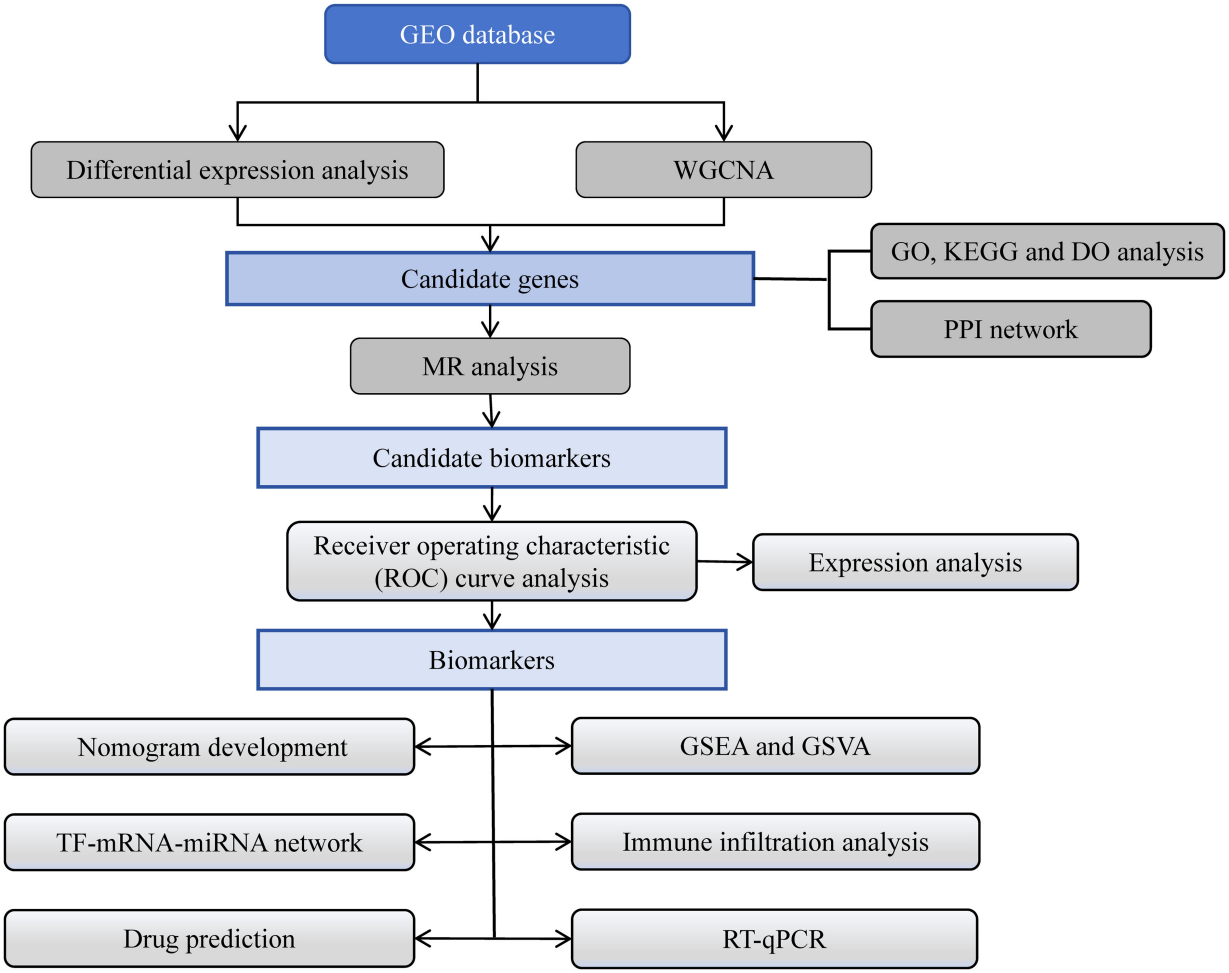
The flowchart of entire analysis process.

We hypothesize that certain genes, which are biomarkers, exert a pivotal influence on the progression of DR. In this study, differential expression analysis, weighted gene co-expression network analysis (WGCNA), functional annotation analysis, protein-protein interaction (PPI) network construction, and MR analysis of data related to DR in the Gene Expression Omnibus (GEO) and the Integrative Epidemiology Unit (IEU) Open genome-wide association study (GWAS) databases identified three biomarkers with a causal relationship with DR. Furthermore, functional enrichment analysis, immune infiltration analysis, regulatory network construction, and drug prediction were performed, and the expression levels of these biomarkers were verified in the two datasets by RT-qPCR. Finally, a diagnostic nomogram was constructed, which could provide new insights into the diagnosis and treatment of DR. The flowchart illustrating the entire analysis process was depicted in **Figure 1**.

## 2 Materials and methods

### 2.1 Data source

The GEO database (https://www.ncbi.nlm.nih.gov/) was used to acquire mRNA expression profile of the GSE221521 and GSE189005 datasets with the GPL24676 and GPL23126 platforms, respectively. The study included 41 and 50 venous blood samples of DR and normal samples, respectively, in GSE221521 as the training set. GSE189005 consisted of 10 and 9 venous blood samples of DR and normal samples, respectively. The IEU OpenGWAS database (https://gwas.mrcieu.ac.uk/) was employed to obtain the GWAS ID and data for exposure factors and DR. DR was considered as the outcome (finn-b-DM_RETINOPATHY). The finn-b-DM_RETINOPATHY dataset comprised of 14,584 DR samples and 16,380,459 single nucleotide polymorphisms (SNPs).

### 2.2 Differential expression analysis

In the GSE221521 dataset, DR-differentially expressed genes (DEGs) between DR and normal samples were identified conducted utilizing the DESeq2 package (v 1.34.0) (|Log2FC| > 0.5, *p* < 0.05) (10). The P value was corrected by Benjamini-Hochberg (BH) method. The volcano map and heat map were drawn employing the ggplot2 (v 3.4.1) and ComplexHeatmap (v 2.16.0) packages, respectively (11, 12).

### 2.3 WGCNA

The WGCNA was implemented to seek DR key module genes. The WGCNA package (v 1.71) was used to perform hierarchical clustering of all samples in the GSE221521 dataset, and outliers were removed (13). Then, the optimal soft threshold (β) was determined by realizing scale-free distribution and setting R^2^ above 0.85. Based on the β value, the module was segmented by applying the standard of hybrid dynamic tree cutting algorithm (deepSplit = 2, mergeCutHeight = 0.3), with each module containing at least 100 genes. Modules with an absolute correlation greater than 0.6 with DR were further analyzed, and genes in these module were DR key module genes.

### 2.4 Identification and functional annotation analysis of candidate genes

The ggVennDiagram package (v 1.2.2) was used to determine the overlap between DR-DEGs and DR key module genes, and candidate genes were identified if there was overlap (14). The clusterProfiler package (v 4.6.0) was used to perform functional annotation analysis, including the Gene Ontology (GO) functions, Kyoto Encyclopedia of Genes and Genomes (KEGG) pathways, and the disease ontology (DO) enrichment analysis (*p* < 0.05) (15). The STRING database (https://string-db.org/) was employed to predict interactions between proteins corresponding to candidate genes with a confidence score threshold of 0.4. The protein-protein interaction (PPI) network was subsequently displayed adopting Cytoscape (v 3.10.0) (16).

### 2.5 MR analysis

The candidate genes served as exposure factors, and DR was used as the outcome for MR analysis. In MR studies, the following three assumptions were made: (a) the presence of a significant correlation between instrumental variables (IVs) and exposure factors is imperative, (b) IVs should not be affected by confounding factors related to exposure factors or outcome, and (c) IVs can affect the outcome only through exposure factors.

The mv harmonize data function in the TwoSampleMR package (v 0.5.6) was used to unify effect alleles and effect sizes (17). Next, SNPs exhibiting significant correlation with candidate genes were selected as IVs (*p*<5×10^-8^), and IVs for linkage disequilibrium (LD) were removed (clump=TRUE, R^2 =^ 0.001, kb=10000). The function extract instruments of TwoSampleMR package (v 0.5.8) was employed for this procedure (17). MR analysis of causality was carried out by five methods—MR Egger, Weighted median, Inverse variance weighted (IVW), Simple mode, Weighted mode), of which results of the IVW were the primary reference (*p*<0.05) (18–22). Scatter plots, forest plots, and funnel plots were prepared to visualize the results. An odds ratio (OR) greater than 1 indicated that the gene was a risk factor for DR, while the value was less than 1, the gene was considered a protective factor.

### 2.6 Sensitivity analysis for MR analysis

The reliability of MR analysis was assessed via sensitivity analysis, consisting of heterogeneity and horizontal pleiotropy tests, as well as Leave-One-Out (LOO) analysis. Initially, the Cochran’s Q test for heterogeneity was conducted, with *p>*0.05 indicating the absence of heterogeneity. Subsequently, a horizontal pleiotropy test was performed by employing the MR pleiotropy test function, with *p>*0.05 indicating that SNPs affected the outcome only through exposure factors. Finally, the LOO analysis was conducted using the MR leave one out function to determine whether a single SNP could significantly alter the overall effects. Genes exhibiting causal relationships with the outcome and passing the sensitivity analysis were identified as potential.

### 2.7 Receiver operating characteristic curve analysis and nomogram development

The pROC (v 1.18.0) (23) package was employed to plot ROC curves of potential biomarkers in the GSE221521 and GSE189005 datasets, and genes exhibiting area under the curve (AUC) values greater than 0.7 were considered reliable biomarkers. Subsequently, the biomarker expression levels in the GSE221521 and GSE189005 datasets were validated. Based on the expression of biomarkers, the rms package (v 6.5.0) was used to develop a diagnosis nomogram for DR patients (24). Calibration curves and ROC curves were constructed to evaluate the accuracy and reliability of the nomogram predictions. The calibration curve was plotted using the calibrate function and boot method.

### 2.8 Gene set enrichment analysis and gene set variation analysis

Using c2.cp.kegg.v2023.1.Hs.symbols.gmt as the background gene set, GSEA of biomarkers was conducted with R clusterProfiler (v 4.6.0) (*p*<0.05) (15). Based on biomarker expression, all samples in the GSE221521 dataset were reorganized into high and low expression groups. The GSVA package (v 1.46.0) was used for GSVA of biomarkers in these two groups (25).

### 2.9 Immune infiltration analysis

The single sample GSEA (ssGSEA) algorithm was used to assess the abundance of 28 immune cells in the samples of DR and control groups. The Wilcoxon test was used to compare the difference in immune cell infiltration between the two groups (p<0.05). The Spearman correlation coefficient between distinct immune cells, as well as between biomarkers and these cells was computed (|r|>0.3, p<0.05).

### 2.10 Construction of regulatory network and drug prediction

The miRNA targeting biomarkers were predicted using the miRNet database (https://www.mirnet.ca), while the transcription factors (TFs) regulating biomarkers were obtained from the networkanalyst database (http://www.networkanalyst.ca). Cytoscape (v 3.10.0) was then used to construct a regulatory network involving TF-mRNA-miRNA interactions (16). In order to explore potential drugs for the treatment of DR, targeted drugs for biomarkers were searched based on Drug Signatures Database (DSigDB) database (http://tanlab.ucdenver.edu/DSigDB), and results with *p* < 0.05 were selected.

### 2.11 Reverse transcription-quantitative polymerase chain reaction

Peripheral venous blood samples were collected from 20 patients in the Second affiliated Hospital of Kunming Medical University, including 10 patients with DR and 10 patients without DR. These samples were divided into two parts, each comprising of five DR samples and five control samples, and RT-qPCR was performed on each part to verify the screened biomarkers. This experiment was approved by the Institutional Review Board of the Second affiliated Hospital of Kunming Medical University (Review -PJ- Research -2024–134). TRIzol (Ambion, Austin, TX) was used to separate total RNA from 10 samples following the manufacturer’s instructions. The concentration of RNA was extracted using NanoPhotometer N50 (IMPLEN GmbH), and the purity of RNA was assessed by measuring the ratio of A260/A280. The SureScript-First-strand-cDRA-synthesis-kit (Servicebio, Wuhan, China) was used for reverse transcription of total RNA into cDNA as per the manufacturer’s instructions. The temperature was set to 25°C for 5 minutes, followed by 50°C for 15 minutes, then raised to 85°C for a duration of 5 seconds. Finally, maintain the temperature at 4°C during reverse transcription. Subsequently, qPCR analysis was performed using the 2xUniversal Blue SYBR Green qPCR Master Mix (Servicebio) according to the provided manual. The amplification conditions were 95°C for 1min, 95°C for 20s, 55°C for 20s, and 72°C for 30s. The primer sequences for PCR are listed in **Supplementary Table 1**. Gene expression levels were normalized to GAPDH as an internal reference and calculated using the 2−ΔΔCq method (26). The characteristics of patients are listed in **Supplementary Table 2.**

### 2.12 Statistical analysis

R software (v 4.2.1) (https://www.R-project.org/.) was utilized to process and analyze data. Statistical analysis was performed using the wilcox.test method in R.

## 3 Results

### 3.1 Acquisition of candidate genes

**Figure 2:**
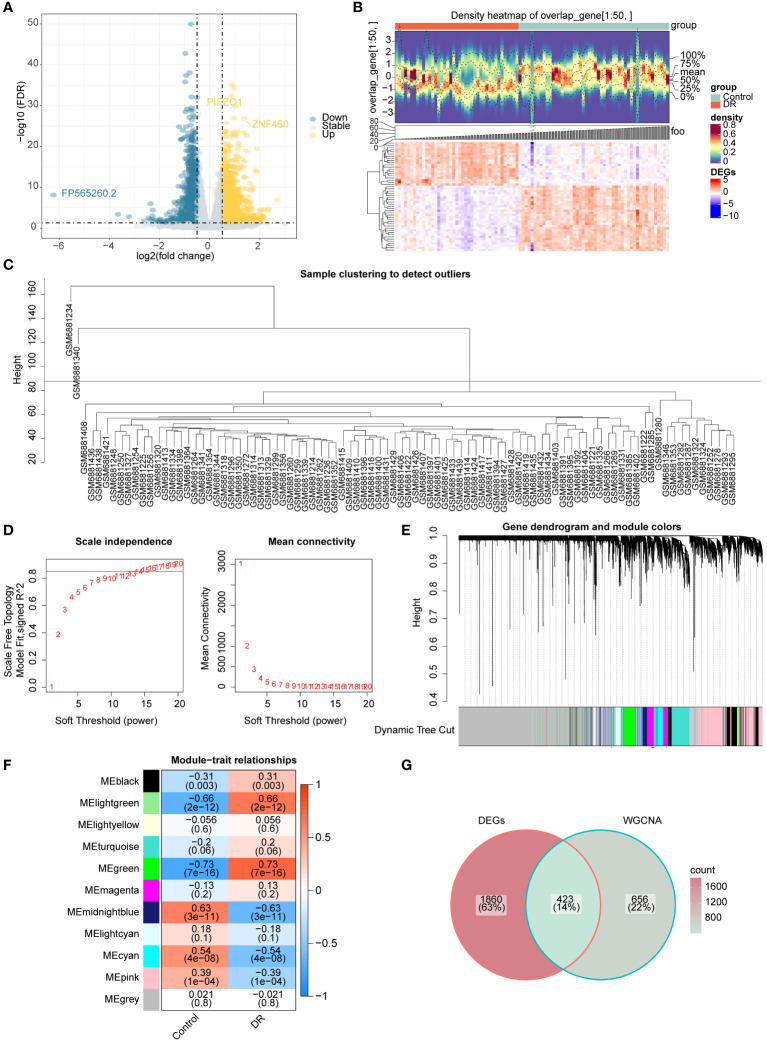
Acquisition of candidate genes **(A)** Volcano map of DR-DEGs **(B)** Heat map of DR-DEGs **(C-F)** WGCNA **(C)** Sample clustering diagram **(D)** The selection of soft threshold β **(E)** Module clustering diagram **(F)** the relevance heat map of gene modules and DR **(G)** Venn map of candidate genes.

The differential expression analysis between DR and normal samples in GSE221521 yielded 2,283 DR-DEGs, comprising 1,304 upregulated DR-DEGs and 979 downregulated DR-DEGs. The volcano map illustrates both upregulated and downregulated DR-DEGs (**Figure 2A**). The heatmap displays the expression of the top 50 DR-DEGs, ranked based on *p*.adj values, in the two groups (**Figure 2B**).

Sample clustering analysis revealed two outliers (GSM6881234 and GSM6881340) in the GSE221521 dataset and were removed for subsequent analysis (**Figure 2C**). The β value was determined to be 13 by setting a threshold of scale-free R^2^ above 0.85 to construct gene modules (**Figure 2D**). As a result, 10 modules were obtained by the hybrid dynamic tree cutting algorithm (**Figure 2E**). Subsequently, the correlation between the green (R=0.73), light green (R=0.66), and midnight blue (R=0.63) module genes with DR were greater than 0.6, and a total of 1,079 DR module genes were obtained from these modules (**Figure 2F**). Finally, 423 candidate genes were determined based on interaction between 2,283 DR-DEGs and 1,079 DR module genes (**Figure 2G**).

### 3.2 Enrichment analysis of candidate genes

**Figure 3:**
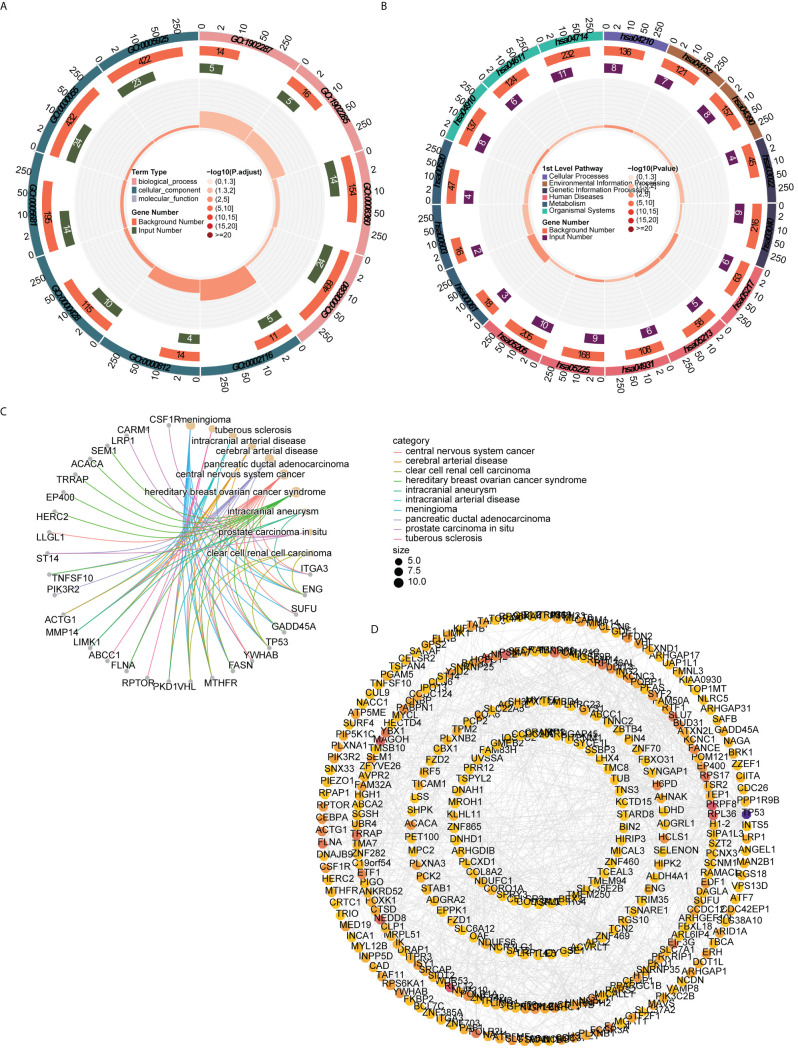
Functional enrichment analysis and PPI network **(A)** GO functions enriched by candidate genes **(B)** KEGG pathways enriched by candidate genes **(C)** Disease enriched by candidate genes **(D)** PPI network of candidate gene.

In order to understand the functions, diseases, and pathways of candidate genes, enrichment analysis was performed. The GO functions of candidate genes included semaphorin receptor complex, semaphorin-plexin signaling pathway involved in axon guidance, and regulation of cell shape (**Figure 3A**). The KEGG enrichment analysis showed that candidate genes were markedly enriched in endometrial cancer, basal cell carcinoma, fatty acid biosynthesis, and apoptosis (**Figure 3B**). The DO enrichment analysis showed that candidate genes were significantly related to meningioma, tuberous sclerosis, intracranial arterial disease, and cerebral arterial disease (**Figure 3C**).

The PPI network constructed to investigate the interactions among genes contained 324 nodes and 815 edges. *AGO2* had a direct interaction with 18 genes (e.g., *CLCN6*, *UBR4*, *SIDT2*), whereas *ACTG1* interacted with 14 genes (e.g., *SRCAP*, *ITGA3*, *HCLS1*) (**Figure 3D**).

### 3.3 Candidate biomarkers exhibiting significant causal relationships with DR

The 423 candidate genes served as exposure factors, with 176 genes exhibiting SNPs, and DR was used as the outcome for the MR analysis. The IVW method was used to identify 13 candidate biomarkers (*OSER1*, *HIPK2*, *DDRGK1*, *PCK2*, *IK*, *IRF5*, *COLGALT1*, *TRPM2*, *SLC38A10*, *TSNARE1*, *PAQR7*, *ZNF142*, and *ARID1A*) that exhibited significant causal relationships with DR (*p*<0.05). Among them, eight candidate biomarkers (*OSER1*, *HIPK2*, *DDRGK1*, *PCK2*, *TRPM2*, *SLC38A10*, *TSNARE1*, and *ZNF142*) exhibited an OR greater than 1 and identified as risk factors for DR. Conversely, five candidate biomarkers (*IK*, *IRF5*, *COLGALT1*, *PAQR7*, and *ARID1A*) demonstrated an OR less than 1 and were considered protective factors for DR (**Table 1**).

**Table 1:** MR analysis results (IVW).

outcome	exposure	gene symbol	Method	P value	OR
finn-b-DM_RETINOPATHY	eqtl-a-ENSG00000132823	OSER1	IVW	0.04	1.052
	eqtl-a-ENSG00000064393	HIPK2	IVW	0.04	1.259
	eqtl-a-ENSG00000198171	DDRGK1	IVW	0.022	1.064
	eqtl-a-ENSG00000100889	PCK2	IVW	0.002	1.078
	eqtl-a-ENSG00000113141	IK	IVW	0.039	0.926
	eqtl-a-ENSG00000128604	IRF5	IVW	0.003	0.925
	eqtl-a-ENSG00000130309	COLGALT1	IVW	0.002	0.906
	eqtl-a-ENSG00000142185	TRPM2	IVW	0.043	1.09
	eqtl-a-ENSG00000157637	SLC38A10	IVW	0.027	1.067
	eqtl-a-ENSG00000171045	TSNARE1	IVW	0.0003	1.185
	eqtl-a-ENSG00000182749	PAQR7	IVW	0.003	0.893
	eqtl-a-ENSG00000115568	ZNF142	IVW	0.043	1.094
	eqtl-a-ENSG00000117713	ARID1A	IVW	0.022	0.942

In the scatter plot, the effect of SNPs of *OSER1*, *HIPK2*, DD*R*GK1, *PCK2*,*TRPM2*, *SLC38A10*, *TSNARE1*, and *ZNF142* on DR were positively correlated overall, while the effect of SNPs of *IK*, *IRF5*, *COLGALT1*, *PAQR7*, and *ARID1A* on DR were negatively correlated overall. These results confirmed the above conclusions (**Supplementary Figure 1**). Consistent with the previous results, the forest map illustrated that the MR effect size of risk factors for DR exceeded 0 and the MR effect sizes of risk factor were less than 0, providing further evidence that IVs exhibit no or weak correlation with outcome (**Supplementary Figure 2**). At last, the funnel plot illustrated that MR analysis of 13 candidate biomarkers and DR was consistent with Mendel’s second random law (**Supplementary Figure 3**).

Sensitivity analysis results showed that all 13 candidate biomarkers passed the tests of horizontal pleiotropy and heterogeneity, and LOO analysis affirming the robustness and reliability of our MR analysis (**Supplementary Tables 3**, **4**, **Supplementary Figure 4**).

### 3.4 *OSER1*, *HIPK2*, and *DDRGK1* served as dependable biomarkers

**Figure 4:**
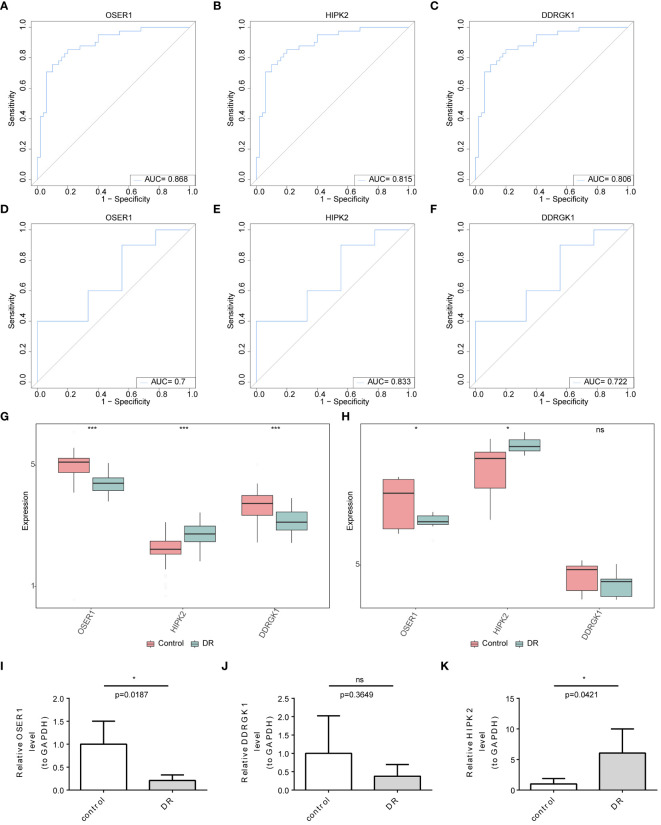
Identification and validation of biomarkers **(A–F)** ROC curve analysis of candidate biomarkers in GSE221521 and GSE189005 datasets **(G, H)** The expression levels of OSER1, DDRGK1 and HIPK2 in GSE221521 and GSE189005 datasets **(I–K)** The expression levels of OSER1, DDRGK1 and HIPK2 in clinical samples by RT-qPCR. *: p < 0.5, ***: p < 0.001, ns: not statistically significant.

**Figure 5:**
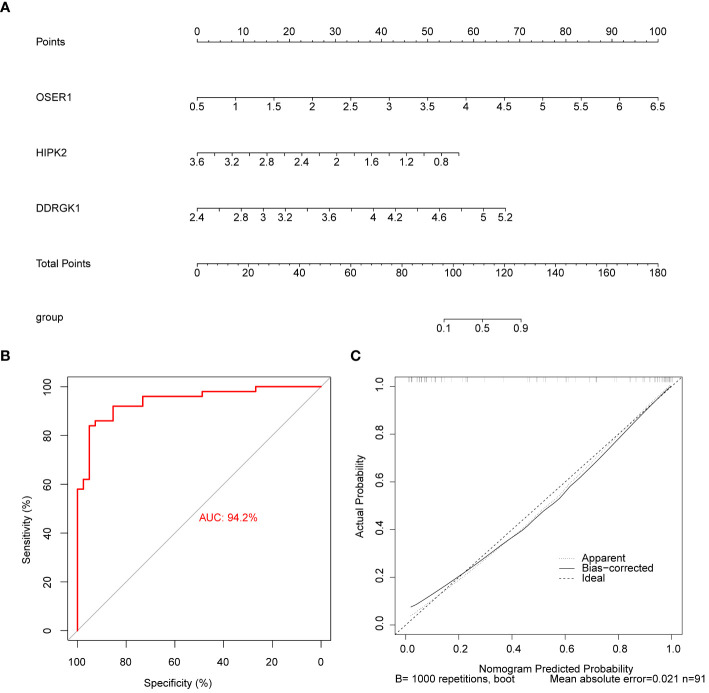
Construction and evaluation of the nomogram **(A)** Construction of the nomogram **(B)** Calibration curve of nomogram **(C)** ROC curve of nomogram.

The AUC values in ROC curves of *OSER1*, *HIPK2*, and *DDRGK1* were 0.868, 0.815, 0.806, respectively, in GSE221521, and 0.700, 0.833, and 0.722, respectively, in GSE189005 (**Figures 4A–F**). Given that all the AUC values exceeded 0.7, these three genes could serve as reliable biomarkers. The expression levels of *OSER1*, *DDRGK1* and *HIPK2* were consistent in GSE221521 and GSE189005 (**Figures 4G, H**). RT-qPCR results also demonstrated a significant high expression of *HIPK2* in patients with DR, whereas *OSER1* exhibited a significant lower expression level (**Figures 4I–K**, **Supplementary Figure S5**). A nomogram was constructed based on the expression levels of *OSER1*, *HIPK2*, and *DDRGK1* (**Figure 5A**). The AUC value for the nomogram was 0.942, and the calibration curve of the nomogram was almost a straight line, indicating a good predictive performance of the nomogram (**Figures 5B, C**).

### 3.5 GSEA and GSVA of biomarkers

**Figure 6:**
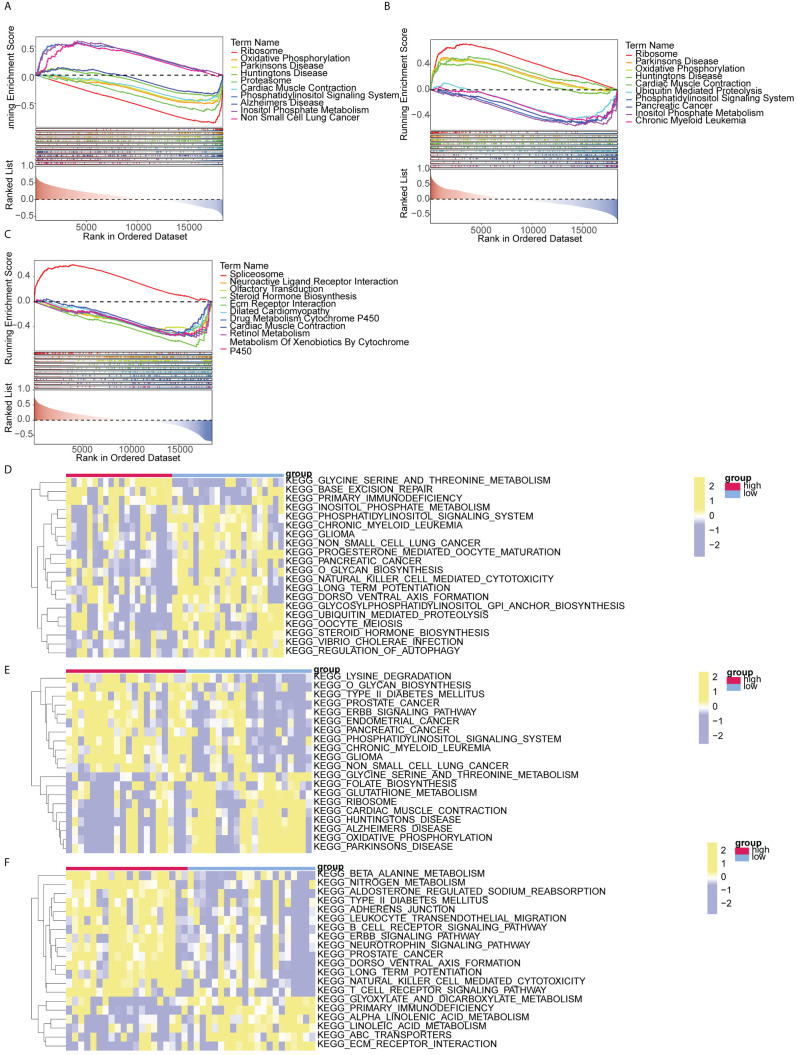
GSEA and GSVA analysis of biomarkers **(A-C)** GSEA analysis of DDRGK1, HIPK2, and OSER1 **(D-F)** GSVA analysis of DDRGK1, HIPK2, and OSER1.

GSEA analysis demonstrated significant associations between *HIPK2* and *DDRGK1* with ribosome, Parkinson’s disease, oxidative phosphorylation, and other pathways. *OSER1* was enriched in spliceosome, neuroactive ligand receptor interaction, and olfactory transduction (**Figures 6A–C**). The top 20 pathways enriched by *DDRGK1*, *HIPK2* and *OSER1* in GSVA are shown in **Figures 6D–F**. The first three pathways (glycine serine and threonine metabolism, base excision repair, etc.) of *DDRGK1* were enriched in the high expression group, while the remaining 17 pathways (such as inositol phosphate metabolism and glioma) were enriched in the low expression group. Similarly, the initial 11 pathways (lysine degradation, prostate cancer, etc.) of *HIPK2* were enriched in the high expression group, whereas the remaining nine pathways (glycine serine and threonine metabolism, ribosome, etc.) were enriched in the low expression group. Among pathways related to *OSER1*, the first 14 (beta alanine metabolism, nitrogen metabolism, etc.) were enriched in the high expression group, and the remaining six (glyoxylate and dicarboxylate metabolism, primary immunodeficiency, etc.) were enriched in the low expression group.

### 3.6 The immune infiltration exhibited notable disparities between the DR and control groups

**Figure 7:**
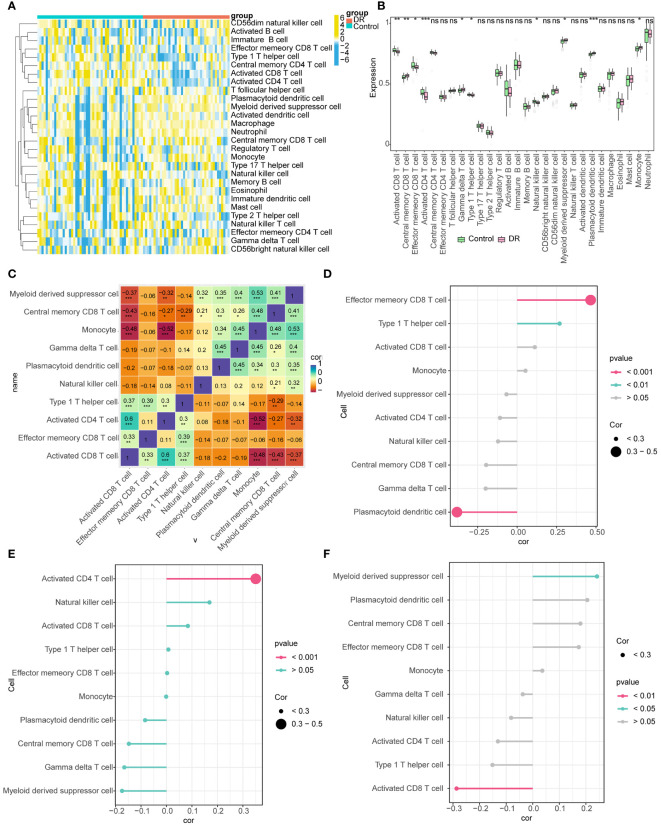
Immune infiltration analysis **(A)** Heat map of the distributions of the 28 immune cells **(B)** Differences in the abundance of immune cells in DR and Control groups **(C)** Differential immune cell correlation heat map (* represents the P-value < 0.05, the number represents the correlation coefficient) **(D-F)** Lollipop chart analysis of correlation between DDRGK1, HIPK2, and OSER1 with differential immune cells (The size of the circle represents correlation, and different colors represent different P-values). *: p < 0.5, **: p < 0.1, ***: p < 0.001, ns, not statistically significant.

The distributions of the 28 immune cells in the sample were visualized using a heatmap (**Figure 7A**). There were 10 immune cell types exhibiting notable disparities between the DR and control groups, such as central memory CD8^+^ T cells, activated CD8^+^ T cells, monocytes, effector memory CD8^+^ T cells, activated CD4^+^ T cells, and myeloid derived suppressor cells (**Figure 7B**). There was a significant positive association between activated CD8^+^ T cells and activated CD4^+^ T cells (R=0.60). Conversely, a significant negative correlation was identified between monocytes and activated CD4^+^ T cells (R=-0.52) (**Figure 7C**). *DDRGK1* expression significantly and positively correlated with effector memory CD8^+^ T cells and negatively with plasmacytoid dendritic cells. Activated CD4^+^ T cells correlated significantly and positively with *OSER1* expression. HIPK2 expression significantly and positively correlated with myeloid derived suppressor cells and negatively with activated CD8+ T cells (**Figures 7D–F**).

### 3.7 Regulatory relationships with biomarkers

**Figure 8:**
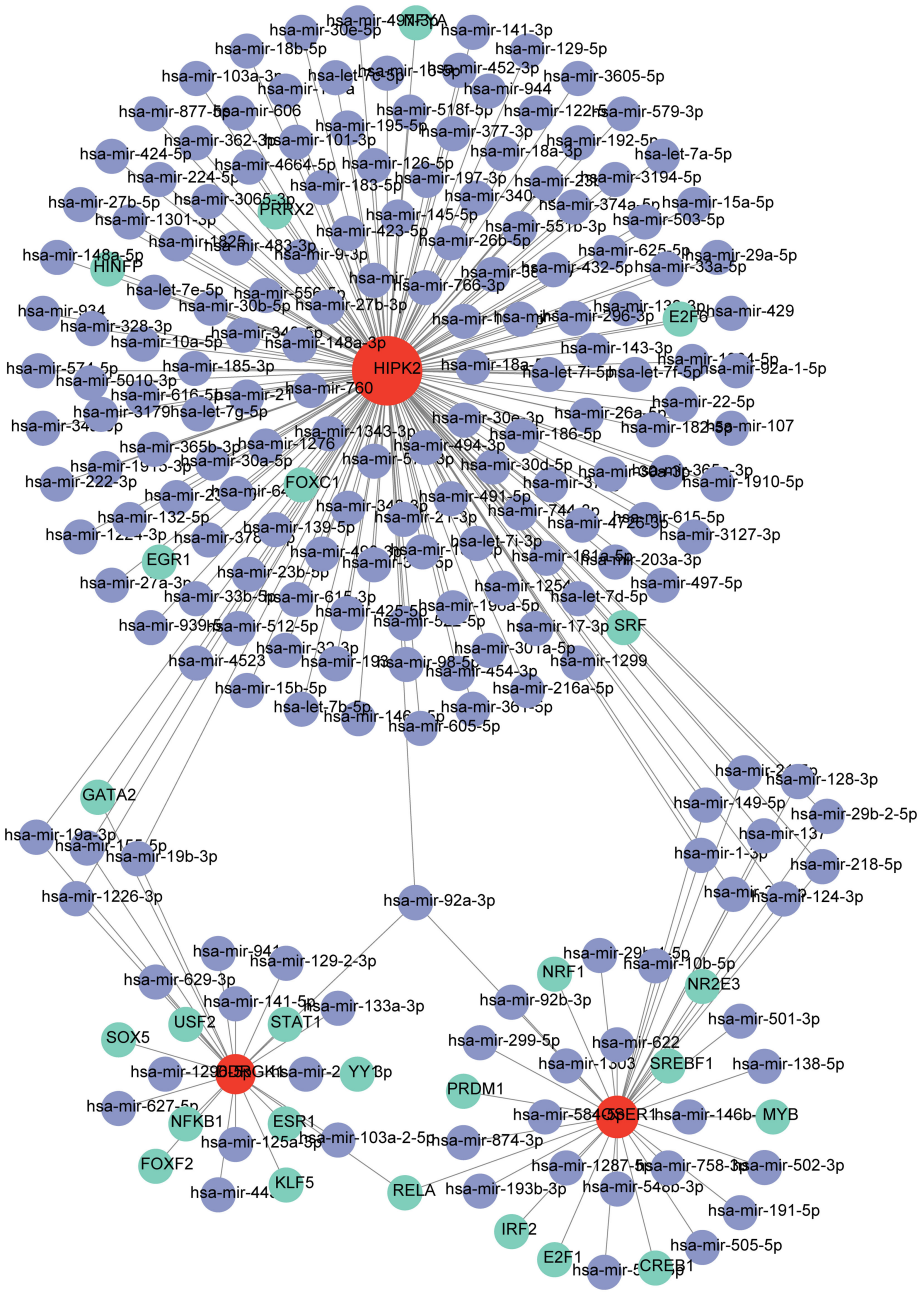
TF-mRNA-miRNA regulatory network (Green represents TF, red represents biomarkers, and purple represents miRNA).

TF-mRNA-miRNA network was composed of 25 TFs, three biomarkers, and 191 miRNAs, with a total of 219 nodes and 233 edges (**Figure 8**). The hsa-mir-92a-3p linked with all three biomarkers. *GATA2* and *RELA* had strong associations with *HIPK2* and *OSER1*, thereby highlighting their role in the pathogenesis of DR. The target drugs of *HIPK2* mainly included GW5074 (Raf1 Kinase Inhibitor I) MRC, LY-317615 Kinome Scan, and GSK650394A MRC. Furthermore, *OSER1* was primarily targeted by Thioguanosine PC3 UP, Elesclomol CTD 00004602, and Gedunin CTD 00003449 (**Table 2**).

**Table 2:** Predicted drugs of biomarkers.

Target genes	Drugs	Chemical formula	Structure
HIPK2	GW5074 (Raf1 Kinase Inhibitor I) MRC	C15H8Br2INO2	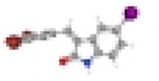
	LY-317615 Kinome Scan	C32H29N5O2	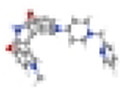
	GSK650394A MRC	/	/
OSER1	Thioguanosine PC3 UP	C10H13N5O4S	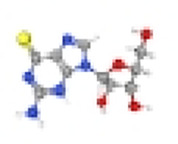
	Elesclomol CTD 00004602	C19H20N4O2S2	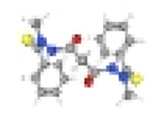
	Gedunin CTD 00003449	C28H34O7	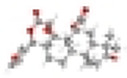

## 4 Discussion

DR is a condition affecting the small blood vessels in the retina, commonly seen in individuals with diabetes. It has emerged as a leading cause of visual impairment among middle-aged people worldwide. Approximately 22.3% of diabetic patients are affected by DR, and about 6.2% experience progressive changes in their retina that can potentially lead to vision loss (27). Timely diagnosis and prompt initiation of treatment can effectively mitigate over 90% of vision loss attributed to DR (28). The occurrence and development of DR are complicated, and its pathogenesis is still unclear. Therefore, it is necessary to investigate further into biomarkers for DR. Bioinformatic analysis techniques, based on the gene expression profiles acquired from databases, have been used to investigate target genes in disease diagnosis (29). For example, inhibition of MAPK3 expression was found through bioinformatics analysis to potentially impact the onset and progression of DR through its regulation of autophagy (30). Likewise, eight potential pyroptosis-related genes involved in the occurrence of DR were analyzed (31). Bioinformatics analysis have allowed us to derive novel insights into the immune mechanisms involved in proliferative diabetic retinopathy, and M2 macrophage-related biomarkers have been recognized to play a role in DR (32). The engagement of hub genes *HMOX1* and *PTGS2*, along with their related TFs and miRNAs, have been shown to potentially play a role in ferroptosis in DR (29).

MR analysis relies on genetic predictors as IVs to investigate the causal association between exposure factors and diseases (33). MR has been used to explore biomarkers of multiple diseases, including diabetes mellitus and DR (34, 35). MR analysis offers crucial evidence regarding the potential causal impacts of numerous alterable exposures, encompassing conventional epidemiological risk factors, lifestyle aspects, and targeted interventions (36).

In this study, based on transcriptome data in the GSE221521 and GSE189005 datasets, candidate genes were identified through differential expression analysis and WGCNA. These candidate genes served as exposure factors, and DR was used as the outcome for MR analysis. A total of 13 candidate biomarkers that exhibited causal relationships with DR were obtained by MR analysis, and the ROC curve demonstrated favorable diagnostic performance of three biomarkers (*OSER1*, *HIPK2*, and *DDRGK1*) for DR. Enrichment analysis delineated pathways associated with the biomarkers, including oxidative phosphorylation, as well as amino acids and glucose. Immune infiltration analysis showed that biomarkers were associated with pro-inflammatory cells such as activated CD4+ T cells or Tfh cells. Moreover, a TF-mRNA-miRNA network was composed of 25 TFs, three biomarkers, and 191 miRNAs, with a total of 219 nodes and 233 edges. Finally, RT-qPCR verified the expression of the biomarkers *in vitro*. Then, we delve into the in-depth discussion of the roles of *OSER1*, *HIPK2*, and *DDRGK1* in DR.

The long noncoding RNA *OSER1* plays a crucial role in the inflammation and apoptosis of rheumatoid arthritis fibroblasts (37), and its low expression was markedly associated with poor survival of cancer patients (38). In the present study, we found that low *OSER1* expression can also contribute to the pathogenesis of DR. Inflammation is a major driver of DR, and *OSER1* is known to promote the inflammatory cascades (37), thereby triggering DR.

*HIPK2* regulates several pro-fibrotic pathways, such as Wnt/β-catenin, liver and cardiac fibrosis, pulmonary, and renal pathways (TGF-β and Notch signaling) (39). *HIPK2* inhibition can result in cardioprotective effects as it would decrease *EGR3* and *CLEC4D* expression levels through ERK1/2-CREB inhibition in cardiomyocytes, as well as through the suppression of Smad3 phosphorylation in cardiac fibroblasts (40). These findings suggest a close link between *HIPK2* expression and fibrosis. Diabetes-associated fibrosis reflects the repair of primary injury and is involved in the pathogenesis of diabetic nephropathy, cardiomyopathy, and liver dysfunction, as well as the development of DR and neuropathy (41). In the present study, we found that *HIPK2* was upregulated in DR patients, suggesting that *HIPK2* may promote the development of DR by promoting the pathological process of retinal cell fibrosis. miR-423–5p is reported to directly bind to *HIPK2*, and its upregulation in DR patients enhances angiogenesis by inhibiting *HIPK2* expression, thereby activating the HIF1α/VEGF signaling pathway (42). This is contrary to the results of the present study, and it is speculated that an increase in the level of VEGF may promote the levels of HIPK2. It is also possible that the discordant result was because our PCR was based on blood samples. In order to clarify the exact relationship between *HIPK2* and VEGF, it is necessary to conduct a study using a larger sample size for the extraction and analysis of vitreal fluids.

*DDRGK1*, a protein containing the *DDRGK* domain, plays a crucial role in the recently identified ufmylation mechanism. Absence of *DDRGK1* leads to significant levels of endoplasmic reticulum (ER) stress (43) and causes a range of conditions, such as malignancies, neurodegenerative disorders, diabetes, and inflammatory disorders (44). Knocking out *DDRGK1* has been observed to trigger ER stress and facilitate apoptosis (45). Due to hyperglycemia and insulin resistance, apoptosis causes DR. Aligning with our finding that low *DDRGK1* expression in DR patients, it is possible that ER stress induced by decreased *DDRGK1* may contribute to the development of DR. However, the large variation in the RT-qPCR results may be related to the small sample size, and more samples are needed for verification of the results.

The present study is the first to demonstrate the association between biomarkers *OSER1* and *DDRGK1* and DR. We found that *HIPK2* may affect fibrosis and VEGF levels through some signaling pathways, suggesting that *HIPK2* **s**erves as reliable biomarkers for DR.

The primary pathophysiological alterations observed in DR are attributed to long-term hyperglycemia, which triggers oxidative stress and inflammation within the microvessels of the retina. Consequently, this leads to thickening of the basement membrane surrounding retinal capillaries, increased permeability of retinal blood vessels, and neovascularization (46). Glycemic control is achieved through the coordinated interaction among glycolysis, the Krebs cycle, and oxidative phosphorylation. Disturbing this equilibrium results in various biochemical and molecular alterations in DR (47). GSEA and GSVA of these three biomarkers provided details of the enrichment between these genes and related pathways. The biomarkers were enriched in pathways of oxidative phosphorylation, possibly indicating that hyperglycemia disrupts the balance between glycolysis and oxidative phosphorylation, leading to other biochemical and molecular changes in retinal cells that have been linked to the neural and microvascular complications of DR. In summary, the expression of these biomarkers may influence the imbalance between glycolysis and oxidative phosphorylation caused by hyperglycemia, potentially leading to retinal microvascular changes associated with DR.

Amino acids play a crucial role in the formation of tissues, specialized proteins, hormones, enzymes, and neurotransmitters. They also contribute to energy metabolism through gluconeogenesis and play important roles in various metabolic pathways (48). Glutamine and arginine levels in the peripheral blood tend to increase in DR patients (49, 50), and methionine level decreases in blood samples of DR patients (48). Therefore, these amino acids can also be used as biomarkers for the early diagnosis of DR. In this study, the pathway of glycine, serine, and threonine metabolism was enriched in the high expression group of *DDRGK1*. Among *OSER1-*related pathways, beta alanine metabolism, nitrogen metabolism, and other amino acid pathways were enriched in the high expression group. Evidence from previous metabolomics studies and our study suggests that metabolic pathways of many amino acids are involved in the occurrence of DR. Consequently, there may be numerous interactions between metabolic pathways of amino acids and glucose associated with the three biomarkers identified in our study, which require further investigation.

Diabetes is a metabolic disorder, and chronic inflammation plays a vital role in the development of type 2 diabetes. We found 10 immune cell types exhibiting notable disparities between the DR and control groups, including activated CD4^+^ T cells. It is well established that CD4^+^ T cells are involved in inflammation (51), and the accumulation of CD4^+^ T cells, B cells, and macrophages is observed in the vitreous of DR patients (52). Inflammation can also affect retinal vascular pathology in DR through the activation of pro-inflammatory retinal microglia by the innate immune system and the transformation of CD4^+^ T cells into pro-inflammatory Th1 and Th17 cells (53). *OSER1* can affect the inflammatory response in DR patients by affecting activated CD4^+^ T cells, leading to the occurrence of DR. The number of a relatively new subgroup of circulating CD4^+^ T cells i.e., follicular helper T (Tfh) cells, which are located at the germinal center, is reported to increase in DR patients compared to healthy individuals (52, 54). The present study also showed that the number of Tfh cells increases in the DR group. These findings further support our results that the number of activated CD4^+^ T cells significantly and positively correlated with *OSER1* expression, suggesting *OSER1* may promote inflammation and accelerate vascular injury, leading to the development of DR through activated CD4^+^ T cells or Tfh cells.

In conclusion, three biomarkers, namely *OSER1*, *HIPK2*, and *DDRGK1*, were found to play a role in DR pathogenesis. The diagnostic nomogram for DR constructed in this study will help further improve the diagnosis of DR. These three biomarkers are expected to become potential targets for the diagnosis and treatment of DR patients. One limitation of the present study is that the results are based on the bioinformatic analysis of a public database, and further experiments are needed to explore the detailed roles of the biomarkers. Additional clinical studies are needed to validate the results. *OSER1* and *DDRGK1* have not been studied earlier in the context of DR, and additional studies are needed to ascertain their role in DR pathogenesis. Our future studies will focus on these two biomarkers. The relationship between *HIPK2* and DR in the present study was found to be opposite of what has been reported earlier; again, further research is needed to explore these pathways.

## Data availability statement

Publicly available datasets were analyzed in this study. This data can be found here: https://www.ncbi.nlm.nih.gov/, https://gwas.mrcieu.ac.uk/.

## Ethics statement

The studies involving humans were approved by Ethics Committee of the Second Affiliated Hospital of Kunming Medical University. The studies were conducted in accordance with the local legislation and institutional requirements. The participants provided their written informed consent to participate in this study.

## Author contributions

JYL: Conceptualization, Methodology, Writing – review & editing. JHL: Conceptualization, Methodology, Writing – review & editing. YT: Conceptualization, Methodology, Writing – original draft. KZ: Formal Analysis, Writing – original draft. XZ: Data curation, Writing – review & editing. JZ: Writing – review & editing. HZ: Conceptualization, Methodology, Project administration, Writing – review & editing.
