# Supplementary material for: Transcriptome analysis combined with Mendelian randomization screening for biomarkers causally associated with diabetic retinopathy
Source: Front Endocrinol (Lausanne). 2024 Jul 3;15:1410066. doi: 10.3389/fendo.2024.1410066 (PMC11251905; doi:10.3389/fendo.2024.1410066)
Supplement: Supplementary file 1 [file DataSheet_1.docx]

Supplementary Material

# Supplementary Tables

**Supplementary Table. 1** Primer sequences for PCR

**Supplementary Table. 2** The characteristics of patients

**Supplementary Table. 3-4** The result of Horizontal pleiotropy and heterogeneity tests.

# Supplementary Figures

**Supplementary Fig. 1** Scatter plot of MR analysis

**Supplementary Fig. 2** Forest maps of MR analysis

**Supplementary Fig. 3** Funnel plots of MR analysis

**Supplementary Fig. 4** The result of LOO analysis

**Supplementary Fig. 5** Supplementary PCR
