# Supplementary figures and images for: Transcriptome analysis combined with Mendelian randomization screening for biomarkers causally associated with diabetic retinopathy
Source: Front Endocrinol (Lausanne). 2024 Jul 3;15:1410066. doi: 10.3389/fendo.2024.1410066 (PMC11251905; doi:10.3389/fendo.2024.1410066)

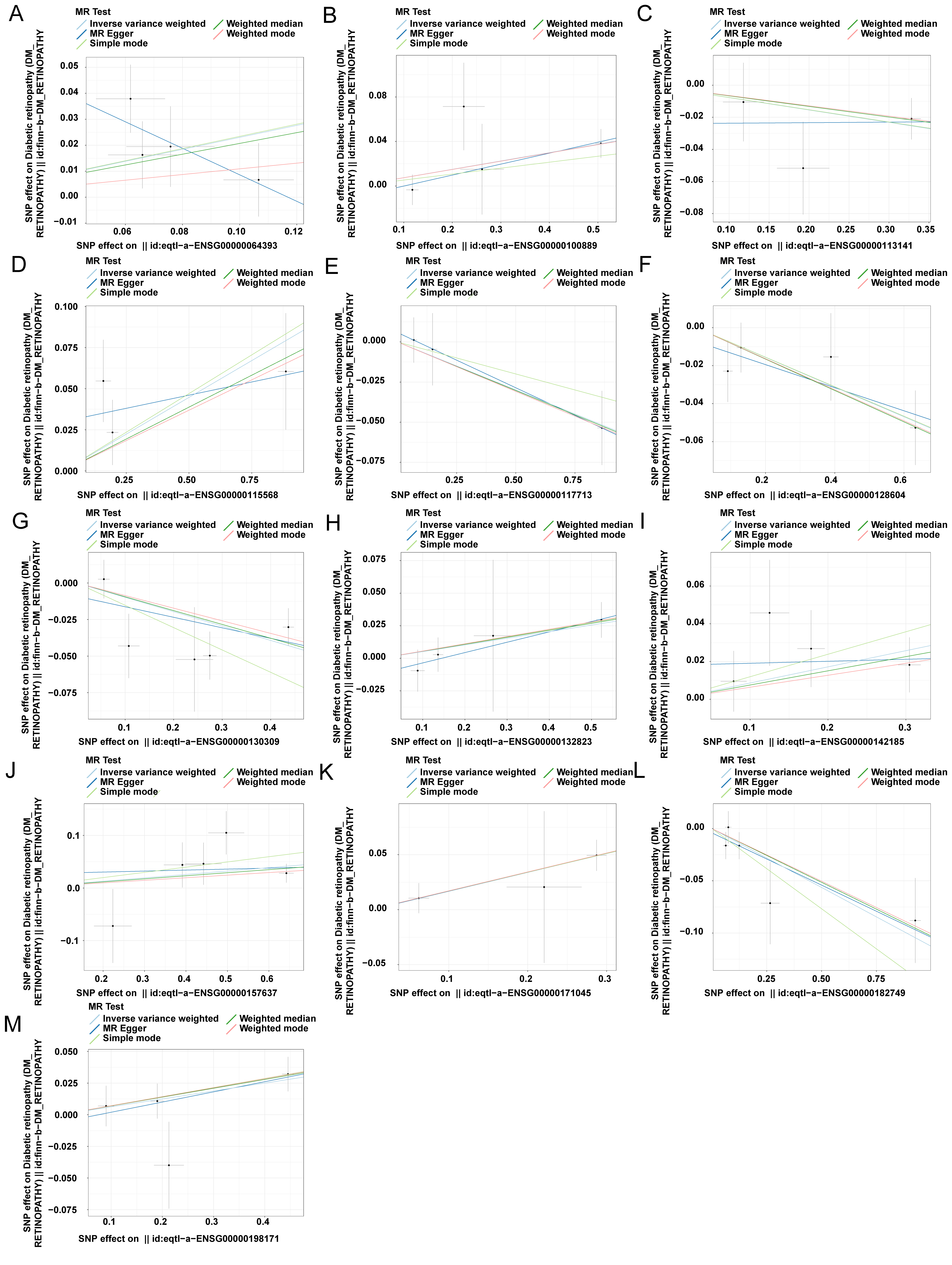

Supplement: Supplementary file 6 [file Image_1.tif]

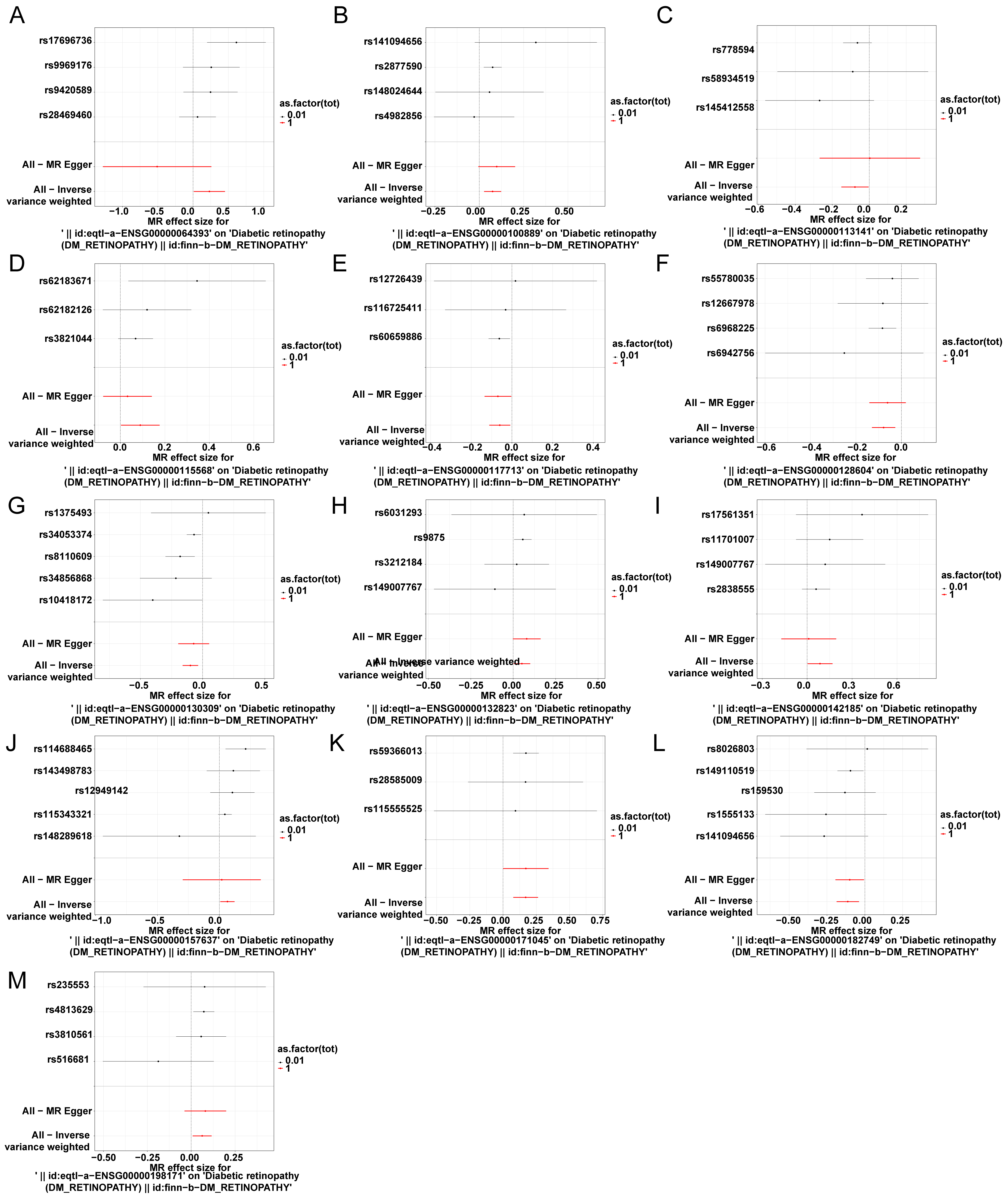

Supplement: Supplementary file 7 [file Image_2.tif]

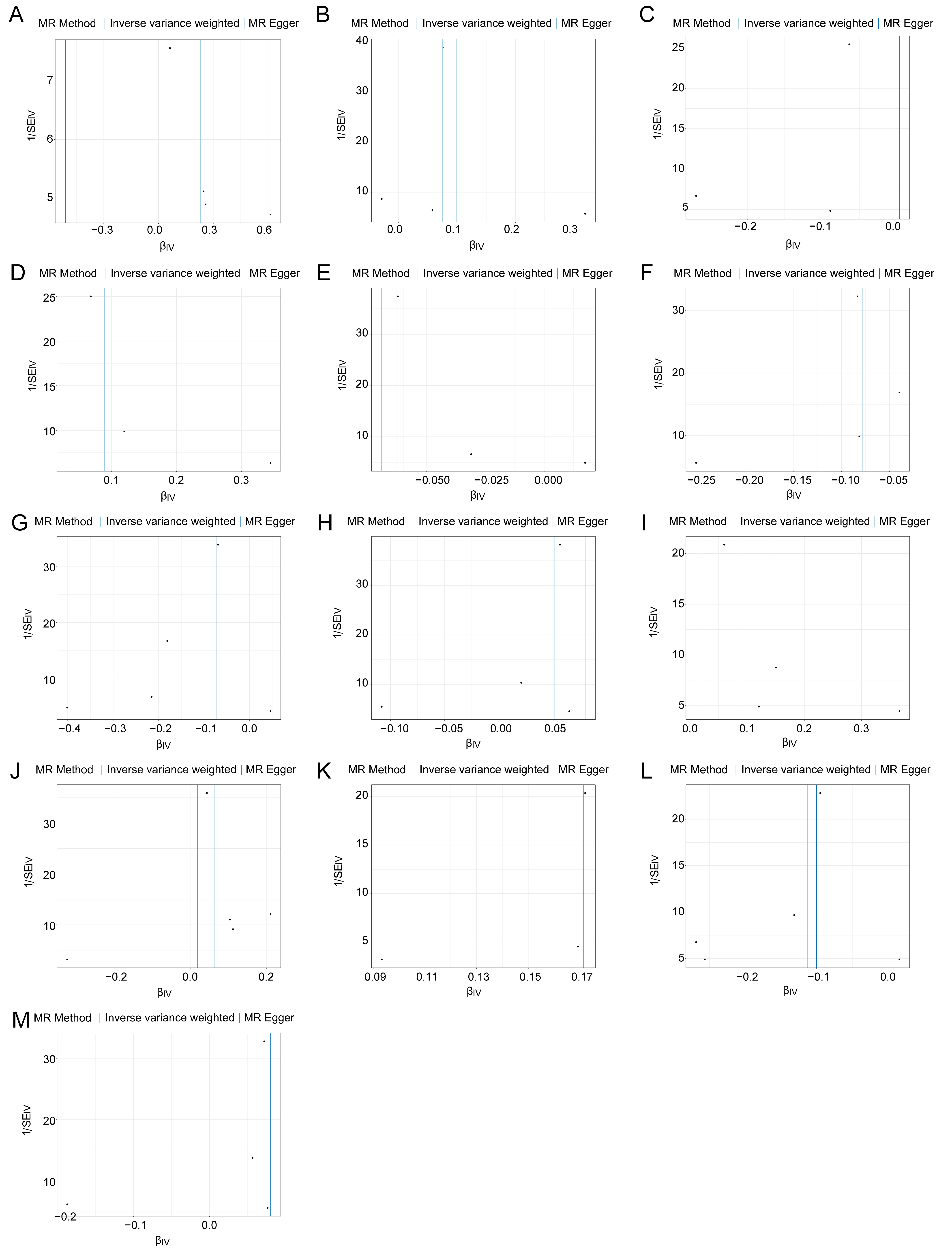

Supplement: Supplementary file 8 [file Image_3.tif]

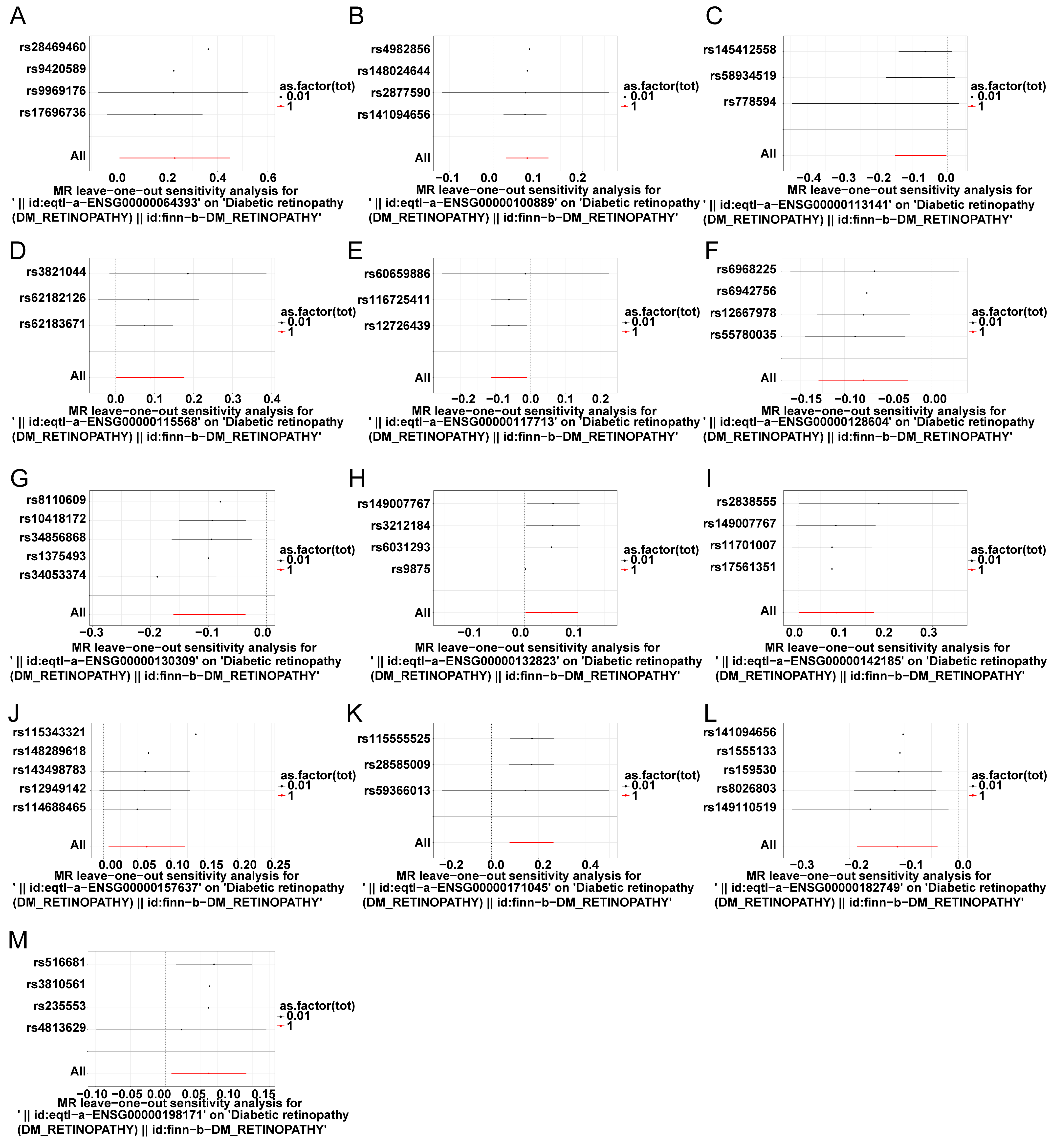

Supplement: Supplementary file 9 [file Image_4.tif]

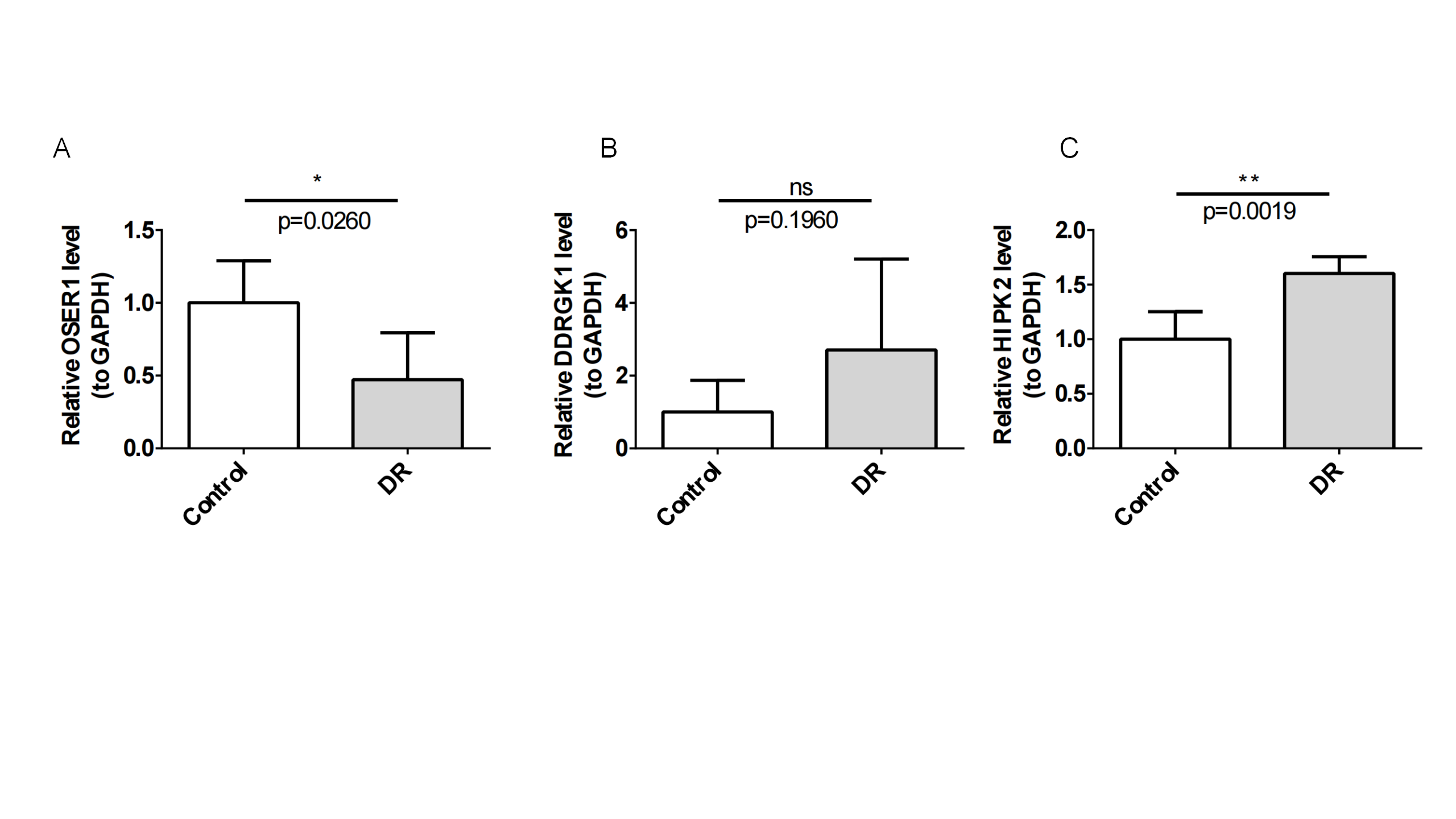

Supplement: Supplementary file 10 [file Image_5.tif]
